# Transient Neonatal Myasthenia Gravis: A Case Report

**DOI:** 10.7759/cureus.20592

**Published:** 2021-12-22

**Authors:** Catarina Santiago Gonçalves, M Ines Nunes Marques, Sónia Antunes, Ana Serrano

**Affiliations:** 1 Pediatrics, Hospital do Espírito Santo de Évora, Évora, PRT

**Keywords:** neostigmine, immunoglobulin, anticholinesterase agents, hypotonia, transient neonatal myasthenia gravis

## Abstract

Myasthenia gravis (MG) in the neonate is usually due to placentally transferred antibodies to the acetylcholine receptor (AChR), resulting in impaired neuromuscular transmission. It occurs in 10%-15% of newborns born to women with MG.

We present a male newborn admitted to the neonatal intensive care unit (NICU) 38 hours after birth due to feeding difficulties and choking episodes. He was born to a mother with MG after an uneventful, well-followed pregnancy. Physical examination revealed a weak cry, persistent inability to fully close his eyelids, weak facial mimic, and a mouth that was always held open with swallowing and sucking difficulties. He assumed a frog leg position and showed generalized hypotonia with marked head lag. No respiratory distress was present. Laboratory evaluation showed an elevated anti-acetylcholine receptor antibody concentration (36.30 nmol/L; normal: <0.25 nmol/L). Transient neonatal myasthenia gravis (TNMG) was admitted, and an anticholinesterase agent was initiated. Given that he showed only a mild clinical improvement, two doses of immunoglobulin were administered on the eighth and ninth days of life. Anticholinesterase agents were progressively reduced and suspended on day 31 of life with clinical improvement. He was discharged home at one month of life clinically asymptomatic. He was evaluated one month later and was doing well.

A positive history of MG in the mother associated with a suggestive physical examination may be sufficient to make the diagnosis of transient neonatal MG, emphasizing the importance of good medical history. With prompt diagnosis and appropriate management, most newborns experience spontaneous remission after a period of weeks to months.

## Introduction

Transient neonatal myasthenia gravis (TNMG) is an antibody-mediated disorder caused by the transplacental transmission of maternal antibodies directed against the acetylcholine receptor (AChR) and, less frequently, muscle-specific kinase (MuSK), resulting in impaired neuromuscular transmission [1,2]. It is a rare disease and may be present in 10%-15% of newborns born to women with myasthenia gravis, either active or, less commonly, in remission [3-5]. The risk of recurrence in subsequent pregnancies is 75% [4,5]. TNMG mainly manifests with hypotonia and poor feeding that, in most cases, resolves spontaneously and progressively in the first two months of life. Respiratory muscles may be involved leading to respiratory distress and failure, requiring ventilatory support. It is a clinical diagnosis in which a suggestive clinical history and physical examination may be sufficient to confirm the diagnosis. Supportive treatment may be enough in mild cases, but severe cases require anticholinesterase agents. We report a case of a newborn with TNMG who needed feeding support and pharmacological treatment.

## Case presentation

A 2990 g newborn male, the second child of non-consanguineous parents, was transferred to the neonatal intensive care unit (NICU) 38 hours after birth due to feeding difficulties and choking episodes. He was born to a 31-year-old G6P2A4 mother after an uneventful, well-followed pregnancy, in which the mother felt normal fetal movements. She was diagnosed with MG nine years before and medicated with pyridostigmine. Thymectomy was performed three years after the diagnosis due to a thymoma. She was asymptomatic during pregnancy. His nine-year-old sister was born just before the mother’s diagnosis and had no symptoms during the neonatal period. She had three spontaneous abortions, and no investigation was undertaken.

**Figure 1:**
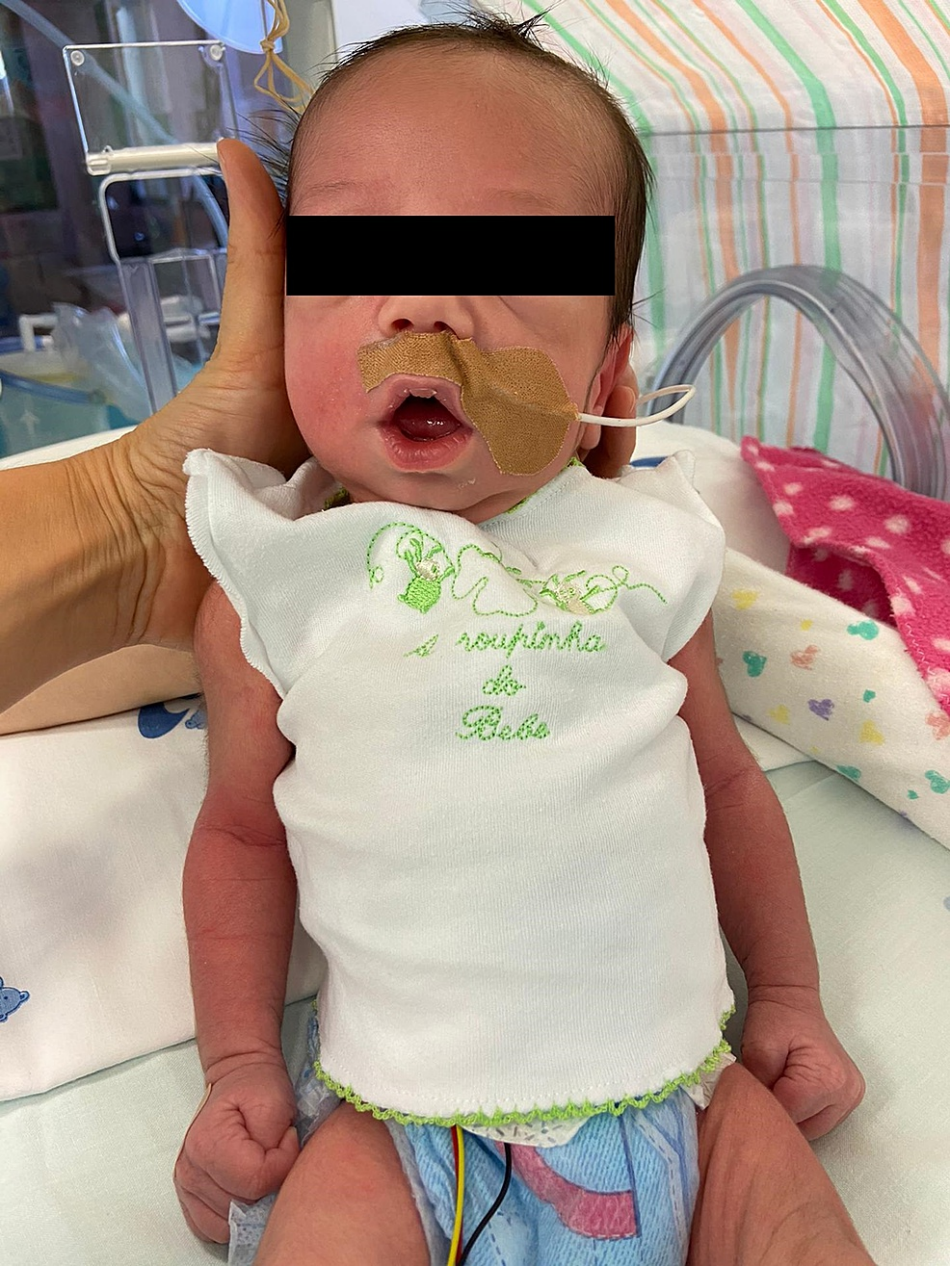
Newborn with a persistently open mouth.

An elective cesarian section, due to a previous cesarian section, was performed at 41 weeks of gestation. The delivery was uneventful, with Apgar scores of 9 and 10 at one and five minutes, respectively. Two hours after birth, he developed intermittent grunting with apparent normal suction reflexes. At first, he was breastfed but rapidly demonstrated feeding difficulties due to poor sucking. Initial laboratory evaluation, including complete blood count, differential count, C-reactive protein, and kidney function, demonstrated normal results, except for elevated creatinine kinase and elevated anti-AChR antibody concentration of 36.30 nmol/L (normal: <0.25 nmol/L). On admission to NICU, he revealed a weak cry, swallowing and sucking difficulties, weak facial mimic, and a constantly open mouth (Figure 1).

**Figure 2:**
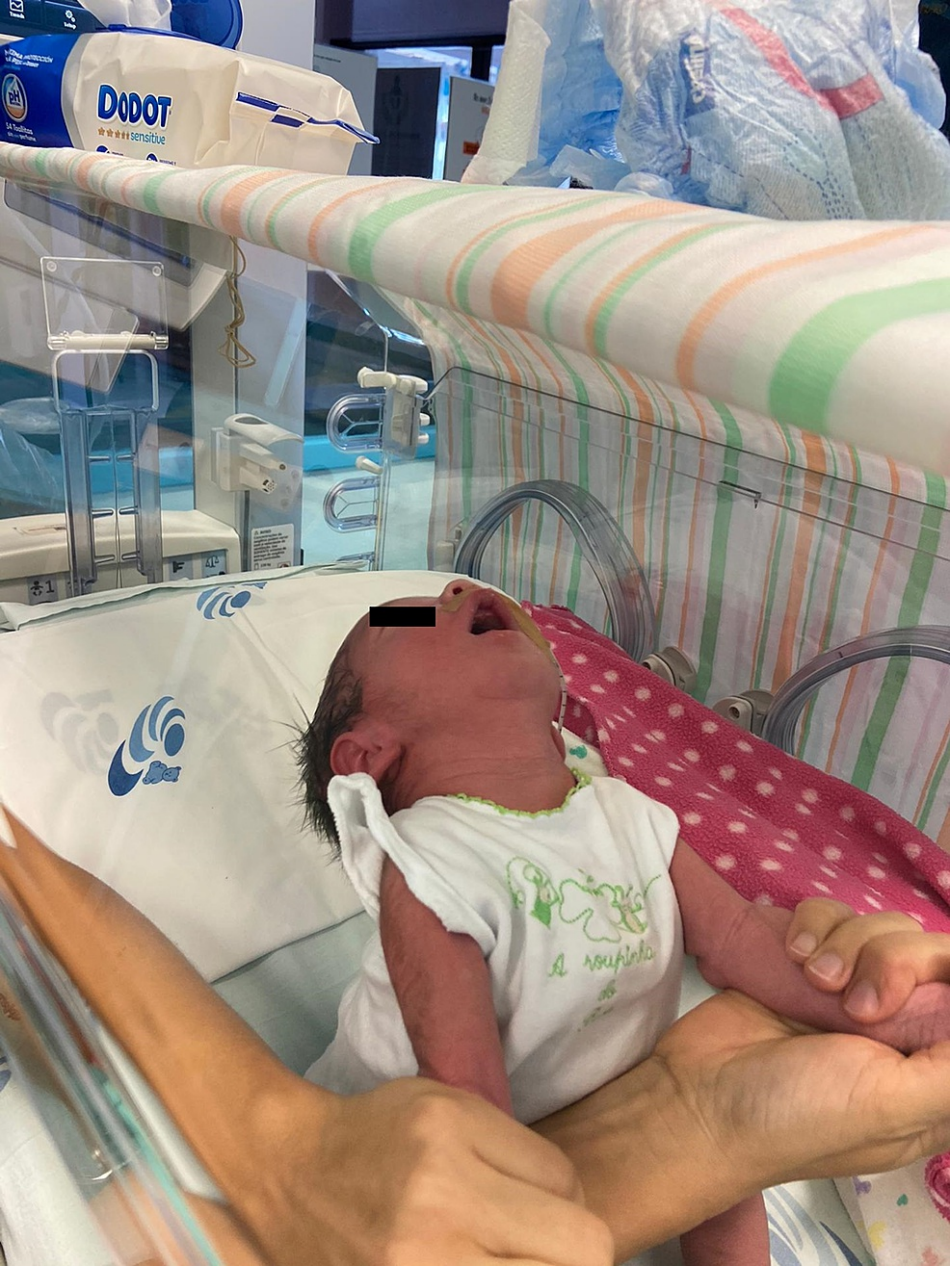
Generalized hypotonia with marked head lag.

He assumed a frog leg position and showed generalized hypotonia with marked head lag (Figure 2).

Ptosis was not present, but there was a persistent inability to fully close his eyelids. No respiratory distress was present. Moro reflex and grasp reflex were present. Due to feeding difficulties, he started gavage feeding.

TNMG diagnosis was admitted, and on his fourth day of life, neostigmine was initiated under neuropediatric guidance at a dose of 0.04 mg/kg, six times a day, subcutaneously (SC). Since there was only a slight improvement in his clinical condition, intravenous immunoglobulin (IVIG) (1 g/kg/day) was administered on the eighth and ninth days of life. On day 12, he showed clinical improvement and started bottle feeding and oral pyridostigmine, 1 mg/kg, four times a day, alternating with neostigmine, which was progressively reduced. On day 14, lacrimation and increased bronchial secretions were noticed with increased feeding difficulties. EKG was normal. A cholinergic crisis was presumed, and anticholinesterase agents were reduced (neostigmine was suspended) with clinical improvement. No other treatment was necessary. On day 22 of life, normal muscle tone and feeding tolerance were present, so pyridostigmine was progressively reduced until suspension on day 31 of life. Three days later, he was discharged home with anti-AChR antibody levels of 5.9 nmol/L.

On follow-up one month later, he was asymptomatic with a normal neurological examination.

## Discussion

TNMG is a self-limited but potentially life-threatening disorder without a prompt diagnosis, adequate respiratory support, and treatment. It is one of the few treatable muscular disorders in newborns. Although the diagnostic evaluation of a hypotonic newborn can be a challenge, a good medical history always helps with the diagnosis, as was with our case. The differential diagnosis of a floppy newborn should include spinal muscle atrophy, neonatal sepsis, central nervous system malformations, infantile botulism, congenital myopathies, chromosomal abnormalities, and others.

A neonate born to a mother with MG is at risk for TNMG. After birth, these patients should be closely monitored by a pediatrician for TNMG signs and symptoms, as symptoms may only begin 3-72 hours after birth [3-5]. Discharge from the hospital should be delayed until clinical stability. Early treatment avoids serious situations that could put the child’s life at risk when severely symptomatic. In our case, symptoms began hours after birth and were initially managed in the maternity ward. He was admitted to NICU only after he had developed severe symptoms.

A positive family history of a mother with known autoimmune MG associated with a suggestive physical examination may be enough to make the diagnosis. Antibody testing is usually highly specific for MG and confirms the diagnosis when positive antibody titers are present. Nonetheless, although AChR antibodies are found in most newborns, their pathogenic role is uncertain because only some infants are symptomatic [6]. For this reason, it is not routinely recommended to measure the antibody levels of a child born from an MG mother.

If MG is suspected, the diagnostic test used to confirm the diagnosis is the infant’s response to acetylcholinesterase inhibitor administration. The most used drug is neostigmine administered intramuscularly (IM) or SC (0.04 mg/kg) [4]. The diagnostic test is positive when clinical improvement is observed approximately 15 minutes after the drug administration and may last one to three hours [7]. If the result is negative or equivocal, another dose may be administered four hours later [5]. Intravenous (IV) administration is not recommended because of an arrhythmia risk, especially before the age of two [5]. IV atropine (0.5 mg/kg) may be needed to control muscarinic side effects, such as diarrhea, abdominal distension, and increased tracheal secretions [4,7]. In this case, there was no immediate clinical improvement after the administration of neostigmine, but the high anti-AChR antibody levels helped confirm the diagnosis.

There are two distinct TNMG clinical forms: typical and atypical. If not adequately supported, the less common, atypical form may present with arthrogryposis, pulmonary hypoplasia, and fetal or neonatal death. Our case is an example of a typical form and is characterized mainly by generalized hypotonia (frog leg position), a weak cry, facial diplegia or paresis, sialorrhea due to inability to swallow, and poor sucking reflex. Ptosis and ophthalmoparesis are less common. They may show little spontaneous motor activity for several days to weeks. Primitive reflexes may be absent or difficult to elicit, but deep tendon reflexes are generally intact. Respiratory muscle involvement may lead to respiratory distress, infections, or apnea. Gavage feeding and assisted mechanical ventilation may be required in severe cases.

Treatment of TNMG includes supporting vital functions, with special emphasis on maintaining adequate ventilation and nutritional status until the weakness spontaneously remits. When the disease takes a more severe course, cholinesterase-inhibiting drugs are the first-line therapeutic agents, being neostigmine the most frequently used. It is administered IM or SC at a dose of 0.04 mg/kg every four to six hours, 30 minutes before meals to improve swallowing [4,5,7]. When dysphagia is improved, it can be given orally (0.5-1 mg/kg), 45 minutes before feeding [4,7]. As clinical improvement is observed, the neostigmine dose can be gradually decreased. In this case, neostigmine was administered SC through the whole course of treatment because it is less painful for the neonate than the IM formulation, and oral formulation is not available in our country. Since there was not a significant clinical improvement after the first few doses of neostigmine, two doses of IVIG were administered (1 mg/kg/day). Although its use is controversial as there is not sufficient evidence to determine its benefits, as with other autoimmune disorders, it is expected that newborns with TNMG may benefit from IVIG [8,9]. In this case, he responded well, and we believe it was due to decreased levels of circulating antibodies, but improvement as part of the natural course of the disease cannot be excluded. Pyridostigmine is an alternative drug to neostigmine with fewer muscarinic side effects and a little longer duration of action [4,5]. An oral formulation is available in our country, and it was started for that reason when the oral route was restored. The average duration of treatment is four weeks, and usually, no other treatment is necessary.

This newborn developed a cholinergic crisis, which usually occurs when the patients are treated with high doses of acetylcholinesterase inhibitors [2,7]. It was characterized by lacrimation, increased bronchial secretions, and feeding difficulties. Diarrhea, abdominal cramps, and, rarely, cardiac arrhythmias may also occur but were not present in this case. A programmed reduction of neostigmine until the suspension was done over three days with symptom resolution. No atropine was needed to control the muscarinic side effects.

Usually, infants regain normal muscle strength as soon as the antibodies disappear from the blood and muscle tissue [1,2]. With prompt diagnosis and appropriate management, most newborns experience spontaneous remission after a period of weeks to months. Complete recovery usually occurs before two months of age in 90% of patients and by four months in the remaining 10% [4,6]. After recovery of TNMG, they are not at increased risk of developing MG in later childhood.

In this case, complete recovery was observed approximately one month after the beginning of the symptoms. Even with a reduced likelihood of neonatal MG after maternal thymectomy, we can verify that the diagnosis of TNMG should always be considered [10,11].

## Conclusions

TNMG, although rare, must always be considered when there are characteristic symptoms and a positive family history of a mother with known autoimmune MG. Discharge from the hospital should be delayed since newborns can become symptomatic until the third day of life. The definitive diagnosis is obtained through clinical response to acetylcholinesterase inhibitors. Measurement of antibody levels is not routinely recommended in asymptomatic neonates born to MG mothers, since there is no correlation between antibody levels and the onset of symptoms. On the other hand, their measurement can be helpful if MG is suspected even if there is no known maternal history since some mothers are asymptomatic. This should be done in symptomatic infants to avoid diagnosis and treatment delay since antibody testing is highly specific for MG. Despite the strict scientific evidence, the use of immunoglobulin may be useful.
